# ST-Elevation Myocardial Infarction Presenting as Acute Limb Ischemia

**DOI:** 10.7759/cureus.10432

**Published:** 2020-09-13

**Authors:** Tamoor Ahmed, Talha Ahmed, Reyaz Haque

**Affiliations:** 1 Internal Medicine, King Edward Medical University/Mayo Hospital, Lahore, PAK; 2 Internal Medicine, University of Maryland Medical Center, Baltimore, USA; 3 Internal Medicine/Cardiovascular Medicine/Interventional Cardiology, University of Maryland School of Medicine, Baltimore, USA

**Keywords:** st-elevation myocardial infarction (stemi), ventricular thrombus, acute limb ischemia

## Abstract

We describe a case of delayed presentation of ST-segment elevation myocardial infarction (STEMI) complicated by ventricular thrombus and peripheral embolization causing limb ischemia. Our patient initially presented with symptoms of acute limb ischemia. However, on preoperative evaluation, STEMI was diagnosed. He required emergent revascularization of the left anterior descending artery followed by thrombectomy of the femoral artery. The cause of the limb ischemia was deemed to be a late presenting STEMI that was complicated by left ventricular thrombus, hence causing lower extremity embolization. Delayed presentations and complications related to STEMI may manifest as acute limb ischemia in the setting of ventricular thrombus formation and subsequent distal embolization.

## Introduction

Left ventricle (LV) thrombus is not an uncommon complication of acute myocardial infarction (MI) and is often associated with systemic thromboembolism. Important risk factors include elderly patients with large anterior MI and those with delayed reperfusion [1]. The incidence may be as high as 15% in patients with ST-segment elevation MI (STEMI) and up to 25% in patients with large anterior MI. Our patient initially presented with symptoms of acute limb ischemia. However, on further preoperative evaluation, it was revealed that he had a late presentation of an anterior STEMI complicated by LV thrombus that had embolized to cause limb ischemia. This was managed by coronary revascularization followed by limb revascularization, and hence salvaging the limb without any perioperative complications.

## Case presentation

**Figure 1:**
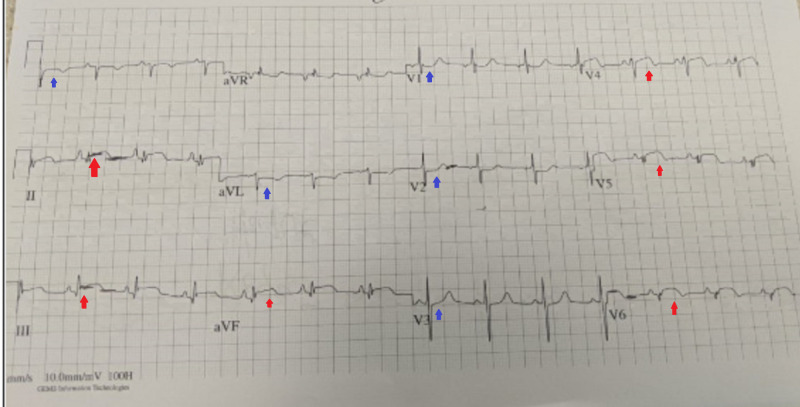
Electrocardiogram on presentation showing ST-segment elevations in leads V4-V6, II, III, and avF (red arrows) with reciprocal changes in leads I, avL, and V1-V3 (blue arrows).

A 55-year-old male presented to the emergency department with numbness and pain in the left lower extremity. The pain started suddenly a few hours ago, was very intense, and associated with a sensation of cold extremity. On presentation, the patient had a heart rate of 68 beats per minute, blood pressure of 128/77 mmHg, respiratory rate of 20 breaths per minute, and a temperature of 98.2˚F. Physical examination revealed the patient to be in distress due to pain and absent dorsalis pedis pulse on the left with pale and cool extremity. Bedside Doppler examination confirmed the absence of dorsalis pedis pulse. A preoperative electrocardiogram (EKG) showed ST-segment elevations in leads V4-V6, II, III, and avF, with reciprocal ST depressions in leads V1-V3, leads I, and avL (Figure 1).

**Figure 2:**
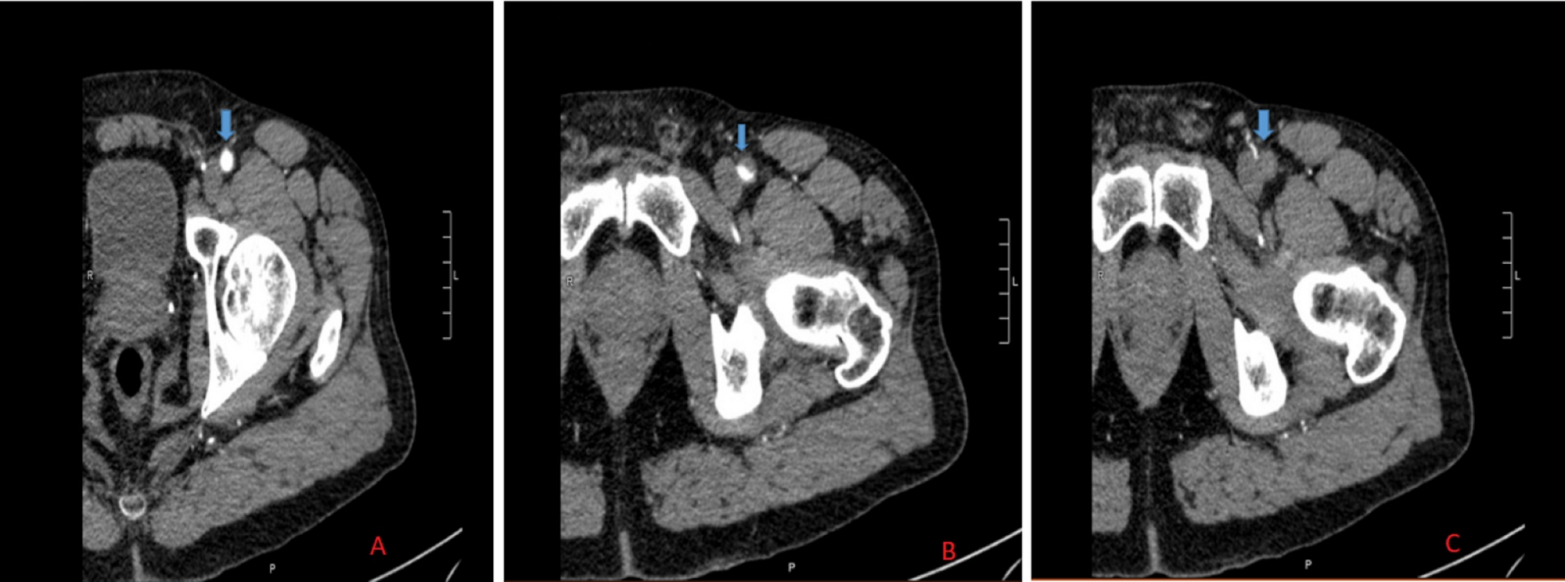
Computed tomography angiogram (CTA) of lower extremities showing acute vascular occlusion at the level of left common femoral artery demonstrated by an abrupt cut-off of the contrast (blue arrows) as shown by successive cross-sectional images from panels A-C.

On further exploration and a thorough history, the patient endorsed intermittent left arm heaviness and tingling over the last two days, but he neglected it and avoided seeking medical care due to the recent coronavirus-related pandemic. Past history was significant for a six pack-year cigarette smoking (quit 35 years ago), well-controlled human immunodeficiency virus (HIV) infection, and depression. Differential diagnoses included acute limb ischemia due to an embolic phenomenon from aortoiliac thrombus or infective endocarditis or cardiac thrombus with embolization and subsequent critical lower extremity ischemia. Complete blood count and basic metabolic panel were unremarkable. Troponin I was elevated to 22.4 ng/mL (normal value < 0.07 ng/mL). Creatine kinase (CK) was elevated at 328 U/L (reference range 55-170 U/L). Coronavirus testing via nasopharyngeal swab was negative. Computed tomography angiogram (CTA) of the left lower extremity showed acute occlusion of the left common femoral artery with diminutive and poorly visualized downstream arterial vasculature (Figure 2).

**Figure 3:**
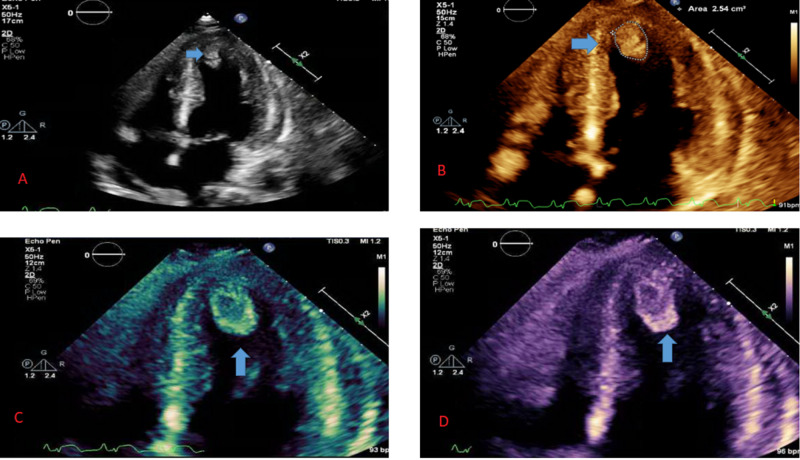
Echocardiogram with apical four-chamber view showing thrombus in the left ventricle, which was likely the source of embolus causing acute limb ischemia (panel A showing apical four-chamber view in gray-scale two-dimensional image and panels B-D showing colored images of the apical view for better visualization of the thrombus which is shown by blue arrows).

The patient was loaded with aspirin 325 mg and clopidogrel 600 mg, and started on intravenous heparin infusion and was emergently taken to the catheterization laboratory. Left heart catheterization (LHC) revealed a subtotal occlusion of the apical left anterior descending artery (LAD) with a mid-systolic bridge. The successful intervention of the distal apical LAD with a 2.0 semi-compliant balloon was performed, hence restoring the flow. Postoperative echocardiogram showed depressed left ventricular ejection fraction (LVEF) of 30% with severe apical and inferolateral wall hypokinesia and an echodensity (2.4 cm x 1 cm) in the LV most consistent with a thrombus (Figure 3).

**Figure 4:**
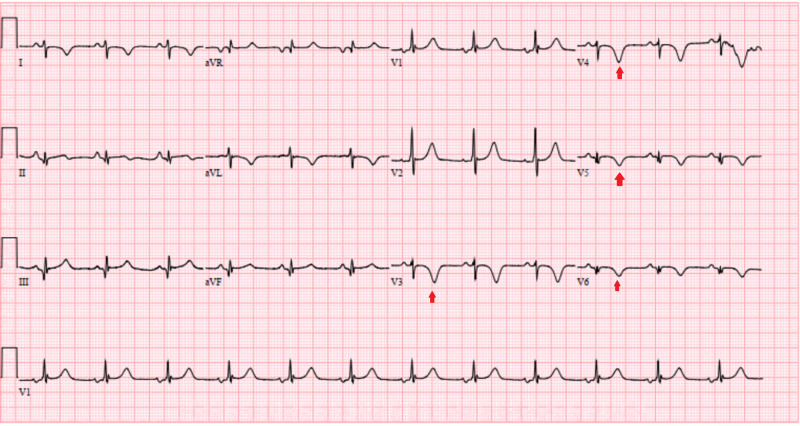
Electrocardiogram after revascularization with persistent T-wave inversions in the leads V3-V6 with resolution of the ST elevations in these leads (red arrows).

Immediately post-LHC, the patient was taken to vascular operation room (OR) on the same day where an open thrombectomy of the left common femoral artery thrombus was performed with the restoration of blood flow in the femoral and downstream vasculature demonstrated by palpable distal pulses. Heparin was bridged with warfarin for the LV apical thrombus, and dual antiplatelet therapy with aspirin and clopidogrel was continued. However, on day 3 of triple therapy (warfarin, aspirin, and clopidogrel), the patient developed a left groin hematoma with a drop in hemoglobin. This required close monitoring for hematoma expansion and blood transfusion. Warfarin and aspirin were discontinued, and the patient was cautiously started on apixaban along with clopidogrel (double therapy). An EKG done after the coronary intervention showed persistent V4-V6 leads and T-wave inversions with resolution of ST elevations (Figure 4).

The initial troponin at presentation was 22.4 ng/mL, which started trending down and was 0.16 ng/mL at day 15 of presentation. The patient was discharged on apixaban and clopidogrel as well as guideline-directed medical treatment with a follow-up surveillance echocardiogram to determine the need for anticoagulation continuation as well as implantable cardioverter-defibrillator placement.

## Discussion

Standard transthoracic echocardiography (TTE) is typically the screening modality of choice for LV thrombus detection and should be performed within 24 hours of admission in those at high risk for apical LV thrombus [2,3]. Our patient had subtle symptoms of arm heaviness that he neglected and did not seek medical care of it. His STEMI was brought to clinical attention late and was complicated by ventricular thrombus formation with embolization leading to acute limb ischemia. One likelihood is that patient’s severe leg pain has made him oblivious of the subtle chest pressure or pain that he otherwise would have noticed in the absence of the leg pain [4,5]. LV apical thrombi have also been associated with systemic thromboembolic events such as stroke leading to significant morbidity and mortality. In rare cases, acute limb ischemia has also been observed requiring emergent surgical management as was seen in our patient [6]. The 2013 American College of Cardiology/American Heart Association (ACC/AHA) STEMI guidelines advise that it is reasonable to add oral anticoagulation (OAC) to dual antiplatelet therapy among patients with STEMI and asymptomatic LV thrombus for three months, targeting a lower international normalized ratio (INR) goal of 2.0-2.5 [7]. The AHA/American Stroke Association 2014 stroke prevention guidelines recommend a similar duration, targeting a higher INR of 2.5 [8]. The European Society of Cardiology 2017 STEMI guidelines advised that once an LV thrombus is diagnosed, OAC should be considered for up to six months, guided by repeated echocardiography and with consideration of bleeding risk and need for concomitant antiplatelet therapy [9]. The optimal duration of OAC in these patients is unclear, and decisions regarding the continuation of OAC should be made on a case-by-case basis. There have been no randomized trials to our knowledge that have compared dual and triple therapy in patients with LV thrombosis complicated by thromboembolism and STEMI [10,11]. Although data from an observational study have shown an increased risk of systemic thromboembolism with the use of OAC as compared to warfarin, prospective randomized studies are lacking in this area [12]. In our case, due to hemodynamically significant groin hematoma requiring transfusion, the treatment rationale was modified to a limited duration of double therapy (apixaban and clopidogrel) with a follow-up echocardiogram to evaluate the necessity for the continuation of anticoagulation.

## Conclusions

Patients with clinically silent or subtle symptoms of STEMI can present with late complications including LV thrombus and its related embolic complications (strokes/transient ischemic attacks, peripheral arterial disease, or mesenteric ischemia), particularly if the presentation is delayed. Physicians should be vigilant in their preoperative examination and evaluation to evaluate the cause of acute limb ischemia in a previously otherwise healthy patient with no history of peripheral arterial disease and otherwise no thromboembolic predisposition. An EKG evidence of STEMI should raise the suspicion of LV thrombus, which is one of the complications of large anterior MI. 
